# Fall Risk Assessment in Stroke Survivors: A Machine Learning Model Using Detailed Motion Data from Common Clinical Tests and Motor-Cognitive Dual-Tasking

**DOI:** 10.3390/s24030812

**Published:** 2024-01-26

**Authors:** Masoud Abdollahi, Ehsan Rashedi, Sonia Jahangiri, Pranav Madhav Kuber, Nasibeh Azadeh-Fard, Mary Dombovy

**Affiliations:** 1 Department of Industrial and Systems Engineering, Rochester Institute of Technology, Rochester, NY 14623, USA; ma8489@rit.edu (M.A.); sj1374@rit.edu (S.J.); pmk2015@rit.edu (P.M.K.); nafeie@rit.edu (N.A.-F.); 2 Department of Rehabilitation and Neurology, Unity Hospital, Rochester, NY 14626, USA; mary.dombovy@rochesterregional.org

**Keywords:** neurological disorder, wearable sensors, motion analysis, fall, neuroscience, TUG

## Abstract

Background: Falls are common and dangerous for stroke survivors. Current fall risk assessment methods rely on subjective scales. Objective sensor-based methods could improve prediction accuracy. Objective: Develop machine learning models using inertial sensors to objectively classify fall risk in stroke survivors. Determine optimal sensor configurations and clinical test protocols. Methods: 21 stroke survivors performed balance, Timed Up and Go, 10 Meter Walk, and Sit-to-Stand tests with and without dual-tasking. A total of 8 motion sensors captured lower limb and trunk kinematics, and 92 spatiotemporal gait and clinical features were extracted. Supervised models—Support Vector Machine, Logistic Regression, and Random Forest—were implemented to classify high vs. low fall risk. Sensor setups and test combinations were evaluated. Results: The Random Forest model achieved 91% accuracy using dual-task balance sway and Timed Up and Go walk time features. Single thorax sensor models performed similarly to multi-sensor models. Balance and Timed Up and Go best-predicted fall risk. Conclusion: Machine learning models using minimal inertial sensors during clinical assessments can accurately quantify fall risk in stroke survivors. Single thorax sensor setups are effective. Findings demonstrate a feasible objective fall screening approach to assist rehabilitation.

## 1. Introduction

Falls are experienced by everyone at least once in their lifetime in the form of sudden traumatic events typically accompanied by a sense of loss of balance. Such events, however, can be a fatal cause of morbidity and mortality in the elderly population, especially those suffering from neurological disorders such as stroke survivors (SS). The US alone sees 800,000 stroke cases (~75% experiencing first time) every year [1]. Stroke is the cause of one out of every 20 deaths and is the fifth leading cause of death in the US [2]. A stroke can affect the neuro-musculoskeletal systems of the body causing sensory, motor, cognitive, and emotional impairments. Such impairments lead to poor quality of life experienced in survivors due to physical (such as paralysis, lack of balance, muscle spasticity, pain, numbness) and mental (memory retention, poor attention span, and difficulty solving problems) limitations. Consequently, the lack of proper motor control increases the risk of fall. The elderly population (>65 yrs.) experiences falls at least once annually, and 10–15% of these falls are fatal [3]. Falls are even more critical in SS by being seven times more prevalent [4]. Furthermore, SS are often prescribed antiplatelet or anticoagulants for secondary stroke prevention, which may cause brain bleeding after experiencing a fall. To prevent fatal injuries, it is critical to identify patients with high fall risk, ensure proper rehabilitation programs to enhance their physical performance, and consequently decrease the risk of fall.

Several efforts have been made to develop scales to evaluate the risk of fall in SS [5,6,7,8]. Examples of such scales include the Fugl–Meyer assessment of motor recovery, Berg Balance Score (BBS), Fall Efficacy Scale (FES), Postural Assessment Scale for Stroke (PASS), and Activity-Specific Balance Confidence (ABC) Scale. Particularly, a large diversity in scales stems from a lack of adequate knowledge about the factors influencing a fall. There are more than 120 factors in the literature as fall risk factors (FRFs) in the stroke community, which can be categorized as subjective and objective. Subjective risk factors are usually measured by a clinician or the subject filling different questionnaires targeting various aspects of the individual’s capability and performance. On the other hand, objective factors are measured using various equipment such as timers, force platforms, optoelectronic cameras, or wearable sensors. Among these, gait and balance analyses using force plates and motion sensor signals are the most popular objective approaches to assessing the risk status. Additionally, recent work has begun exploring the utility of time-dependent spectral descriptors derived from gait sensor data signals to characterize neurological impairments [9,10], which could be later implemented for fall risk assessment as well. Our recent review [11] showed that FRFs that were highly focused upon were age (in 21/27 studies), gender (21/27), motion-related measures (19/27), motor function/impairment (17/27), balance-related measures (16/27), and cognitive impairment (11/27). Among these factors, motion-related measures had the highest rate of significance (i.e., 84% or 16/19). Overall, objective methods are less prone to biases and errors and can provide a more accurate assessment for fall risk.

A total of 18 risk prediction models were proposed in 12 articles as observed from a previous review article [12]. The development of fall-risk scales followed a specific procedure, which started with an initial pool of fall risk factors (FRF), data collection, statistical analysis, and estimating the performance of the algorithm in terms of measures such as accuracy, sensitivity, and specificity. Previous studies on fall risk assessment in the stroke community have considered singular tasks from Activities of Daily Living (ADL) [13,14,15,16]. Cognitive-motor dual-task would provide more realistic information about the functionality of the SS during ADLs. Furthermore, the addition of cognitive loading may help in discriminating between high vs. low fall-risk individuals. The addition of cognitive loading is often overlooked in studies on SS but has been previously considered in a few studies aiming to investigate the effect of dual tasks on the fall risk assessment of patients suffering from Parkinson’s disease and multiple sclerosis [17,18]. Thus, it is necessary to conduct a study having a list of tasks comprising single and dual tasks to identify the effect of the dual tasks on the accuracy of the fall risk assessment models.

In this study, we will develop a new objective model for the assessment of fall risk based on the kinematic features (obtained from wearable motion sensors) specifically tuned for SS during a battery of physical and motor-cognitive dual-tasks (along with clinical information). Furthermore, we will determine the optimal configuration of wearable sensors as well as sub-tasks for the test battery with the minimum number of sensors and physical tasks. Another novelty of our approach is the algorithms that we are going to implement for developing the models. Earlier studies have used traditional statistical tools such as *t*-tests to indicate the significant differences between patients with different fall risk levels. In this study, we have developed a fall-risk assessment model using a Support Vector Machine (SVM), Random Forest (RF), and Logistic Regression (LR) which are among the most well-known classification methods. The overall outcomes of this study may be beneficial to researchers and clinicians alike, working toward developing and improving rehabilitation programs for surviving patients of stroke.

## 2. Materials and Methods

**Figure 1:**
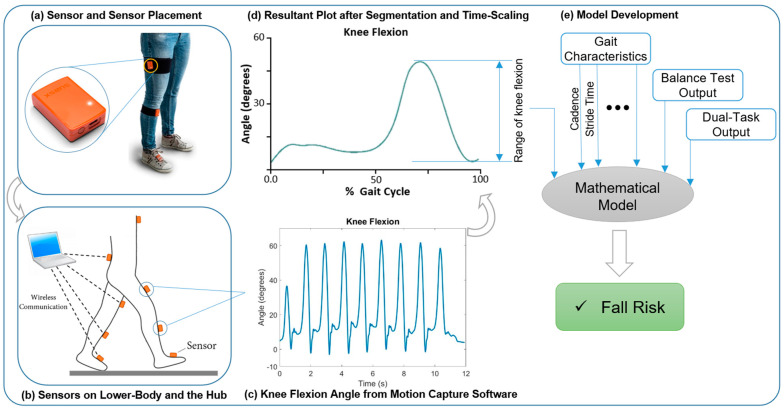
Schematic overview of the study method illustrating a sample signal. Note: this signal was not used in the model development and was only implanted to show the study overview.

An overview of the study methodology is illustrated in Figure 1. This schematic provides a visual representation of the entire research process, with further details and explanations provided in the subsequent subsections.

### 2.1. Participants

A group of 21 stroke survivors were recruited from healthcare systems in the City of Rochester, NY, USA. Participants were included in the study if they (1) had experienced a stroke at least six months prior to the day of the experiment, (2) were able to walk for 10 m with no assistance. Participants were excluded from the study if they suffered from more than three chronic health conditions. Eligible participants were also vetted by clinic directors in addition to the study investigators. The demographics of participants, specifically their gender, age, height, and weight have been collected and considered in the process of fall risk model development. All participants in this study provided written consent according to the best clinical research practices under an approved Institutional Review Board (IRB) process.

### 2.2. Experimental Procedures and Test Battery

For this study, a comprehensive set of physical single and dual tasks was carefully chosen, encompassing a variety of activities, as illustrated in Table 1. These tasks included assessments such as upright stance balance tests, the timed up and go (TUG), the 10 Meter Walk Test (10MWT), and sit-to-stand (STS). Additionally, we introduced motor-cognitive dual tasks for each task in the test battery, where participants were required to perform the designated task while simultaneously counting backward verbally from 200 in increments of 10. In total, the experiment incorporated five single tasks, comprising balance tests with both open and closed eyes, TUG, 10MWT, and STS. Furthermore, five combined motor-cognitive dual tasks were included, resulting in a comprehensive test battery comprising a total of 10 tasks.

### 2.3. Equipment and Tools

**Figure 2:**
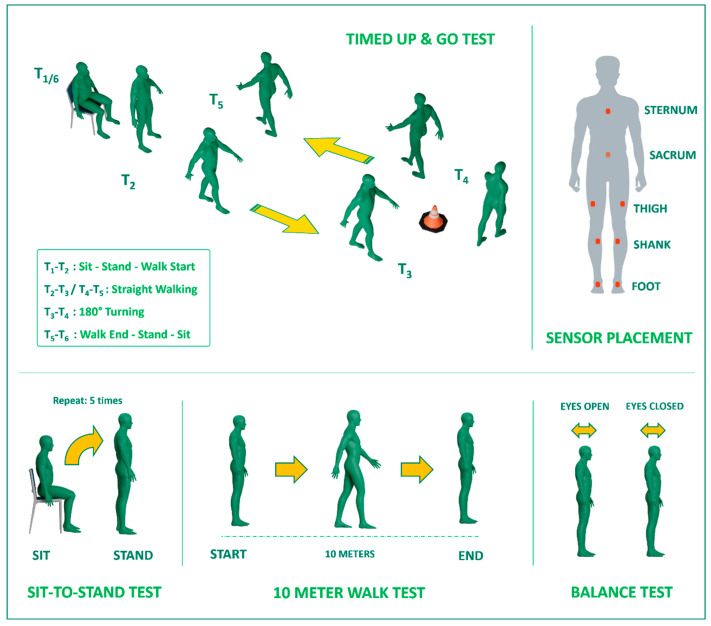
Schematic depicting sensor placement on body segments and the tests.

We used the Movella motion capturing system (Xsens, Enschede, The Netherlands) to acquire the motion data of participants while they performed the tasks. These miniaturized inertial measurement units (IMUs) integrate a 3D accelerometer (range ±6 g), 3D gyroscope (range ±2000 deg/s), and 3D magnetometer (range ±750 mGauss) to capture body segment kinematics. The sensors have dimensions of 47 × 30 × 13 mm and weigh 16 g each. The MTw Awinda units communicate wirelessly via an encrypted proprietary protocol with the MVN Studio software (https://www.movella.com/support/software-documentation, accessed on 15 January 2022) on a Windows computer, enabling untethered monitoring during the clinical assessment tasks. The sensors have a sampling frequency of 60 Hz, which provides adequate capture of human motions along with efficient data bandwidth and storage. Additionally, the large dynamic range of the accelerometer and rate gyroscopes ensures measurement accuracy even for vigorous movements by stroke survivors. To have the minimum number of sensors, which helped in making the experiment more comfortable for the patients, as well as to cover the significant body segments, the 8-sensor configuration was selected for this study. Specifically, the sensors were placed on the feet, shanks, thighs, low back, and sternum of participants, as shown in Figure 2, during the tests. This setup enabled us to have movement data of the entire lower extremity as well as the trunk motion from the upper extremity.

### 2.4. Data Processing and Feature Extraction

In the analysis of kinematic data, a customized MATLAB (https://www.movella.com/support/software-documentation, accessed on 15 January 2022) code was utilized, as detailed in the previous chapter. The kinematic data, which included segment angles, angular velocity, and linear acceleration, underwent initial preprocessing and extraction using the MVN Analyze^®^ software package (Movella, Enschede, The Netherlands, https://www.movella.com/support/software-documentation, accessed on 15 January 2022). To enhance the quality of the data, we applied a low-pass Butterworth filter with a 5 Hz cut-off frequency within MATLAB to the imported data. For each test, we developed a unique MATLAB code to perform segmentation and feature extraction. After executing the code for each trial, all segmentation processes were visually validated. In instances where discrepancies occurred between the code-generated segmentation and the predefined strategy, manual adjustments to segmentation were made.

In the analysis of the TUG test, we divided it into five specific sections, each representing a distinct phase of the task, from standing up from a chair to turning and sitting back down. To identify the starting and ending points of these sections, we defined key events based on signals from body segments. For example, we detected the start of the task (T1) by analyzing signals such as thorax angular velocity. T2 marked the initiation of walking, T3 the start of turning, and so forth. To improve the accuracy of identifying the turning phases, we incorporated the thorax angular velocity in addition to linear acceleration signals from the feet. This detailed breakdown and analysis allowed us to precisely segment the TUG test signals. In the STS test, we focused on angular velocity data to evaluate the transition between sitting and standing positions, particularly in the thighs. By identifying distinct convex shapes in the angular velocity data, we could segment the signals into different phases of the sit-stand-sit activity. This approach provided insights into the duration and patterns of the task. In the 10MWT, we determined heel-strike (HS) and toe-off (TO) events to analyze walking patterns. We used resultant linear acceleration data from shank sensors to identify these events [19]. Subsequently, we calculated various kinematic variables and gait-related factors, including total walk time, cadence, gait speed, and stride duration, providing a comprehensive assessment of participants’ walking performance. From the collected signals in each test, we have extracted several features as shown in Table 2. These features were implemented as the initial pool in the development of the fall risk assessment model.

### 2.5. Machine Learning Model Development

Table 2 illustrates the initial pool of features for the fall risk prediction model, which initially comprised over 92 features. To streamline this feature set and identify the most significant ones, an innovative feature selection process was employed. In the initial step of this approach, a single feature was introduced into the model, and its performance was systematically assessed, with accuracy calculations performed for each individual feature. Moving forward, feature sets were constructed, commencing with the feature that yielded the highest accuracy. Then, the model’s performance, in combination with each of the remaining features, was evaluated by systematically calculating performance values for each pair. The feature set was continuously updated by identifying the pair demonstrating the highest accuracy. This process was consistently maintained, with performance fluctuations in the fall risk model analyzed as each feature was added. Ultimately, the feature set and the model with the maximum accuracy was selected as the baseline model for subsequent analysis. The iterative feature selection process aimed to systematically identify the most relevant feature set. It also involved analyzing performance trends as each feature was incrementally included, all with the ultimate objective of creating a refined predictive model for fall risk assessment.

Three machine learning techniques, namely Support Vector Machine (SVM), Logistic Regression (LR), and Random Forest (RF), were applied to the dataset to distinguish patients with high or low fall risk. As per the literature, these approaches are widely utilized and have demonstrated strong performance in the development of human movement-based models [20]. In order to ensure a bias-free assessment, given the limited dataset of only 21 participants, the “leave one subject out” cross-validation method was employed during the implementation of the machine learning approach. The ‘leave one subject out’ cross-validation approach was systematically utilized to evaluate model performance. This involves iteratively splitting the dataset into a training pool consisting of all except one hold-out test subject. For each iteration, feature selection, hyperparameter optimization, and model training is conducted based on the 20 training subjects. Then the final evaluation is performed by predicting the labels for the excluded test subject. This maximizes the utilization of available data for training, while reserving new subject data for testing to avoid overfitting biases. The procedure repeats until each of the 21 subjects has been individually left out and predicted exactly once. The overall cross-validation performance metrics are then averaged across the 21 hold-out test iterations. Additionally, feature normalization through standardization was utilized in the implementation of the SVM and LR methods. Both normalized and unnormalized features were applied to RF algorithm to assess their performance. Consequently, the discriminative capabilities of each condition were compared by calculating accuracy, sensitivity, specificity.

## 3. Results

Demographic information and clinical characteristics of the participants are presented in Table 3. The study included 21 stroke survivors, with 11 classified as fallers and 10 as non-fallers based on the results of the 6-month follow-ups. The groups were balanced in terms of gender, age, height, weight, and BMI. Most participants reported joint pain (14/21) and feeling unsteady while walking (15/21). More fallers reported being worried about falling while walking (7/11) compared to non-fallers (3/10). Fallers had significantly higher scores on the short FES-I questionnaire assessing fear of falling (13.1 ± 5.1) than non-fallers (9.1 ± 3.0). These results indicate that clinical factors related to balance confidence and steadiness differentiate fallers from non-fallers in our stroke survivor sample.

**Figure 3:**
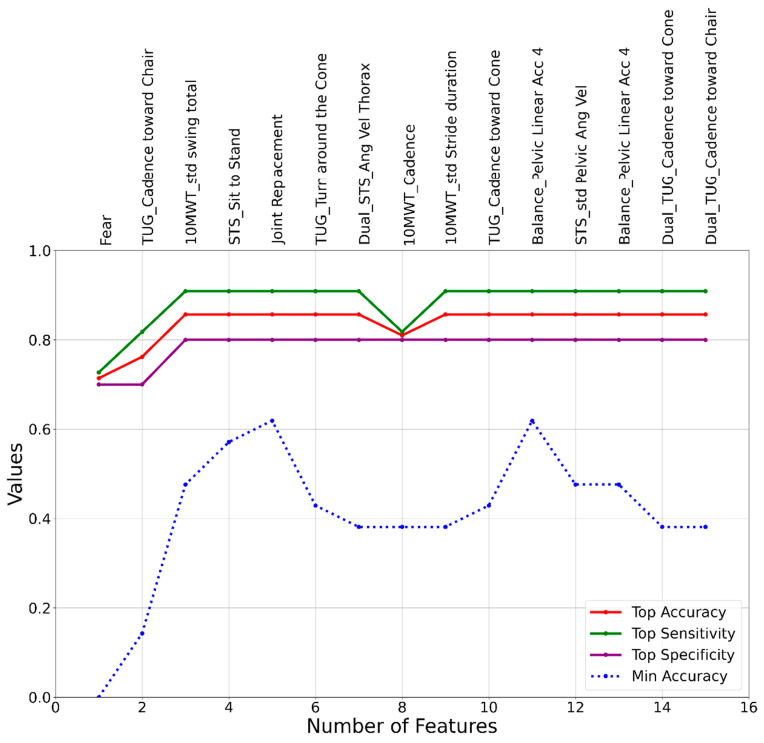
A graphical representation of feature addition order at each step of the heuristic feature selection process for the SVM fall risk assessment model.

**Figure 4:**
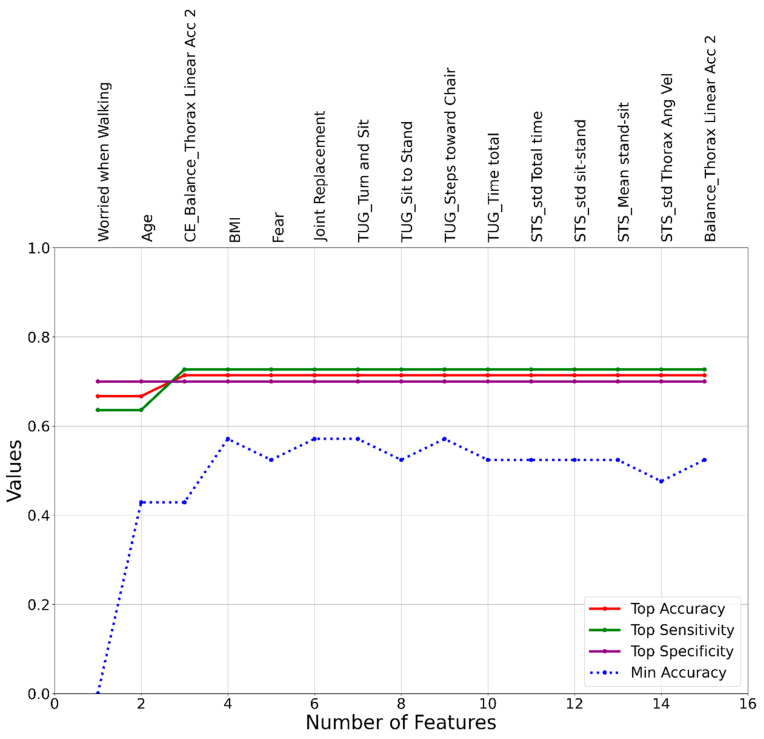
A graphical representation of feature addition order at each step of the heuristic feature selection process for the LR fall risk assessment model.

**Figure 5:**
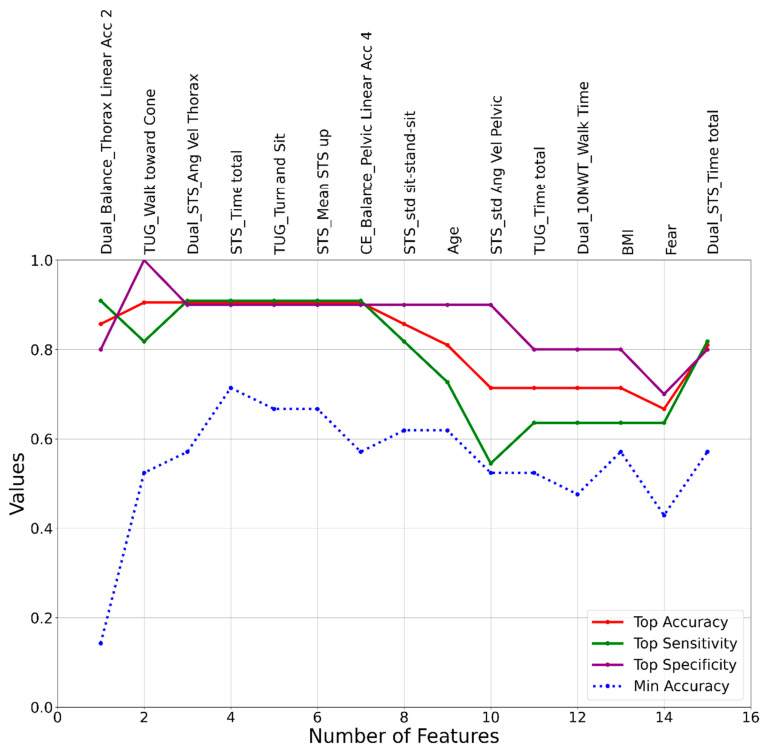
A graphical representation of feature addition order at each step of the heuristic feature selection process for the RF fall risk assessment model.

The outcomes of the machine learning model development were presented in Figure 3, Figure 4 and Figure 5, corresponding to the three different approaches: SVM, LR, and RF. The initial set of features used in the model development process was identical for all cases and consisted of the 92 features listed in Table 2. While our feature space consisted mostly of time domain variables selected a priori based on sensor signals and standard gait metrics, emerging research suggests richer information may be contained within spectral patterns and frequency characteristics of motion sensor data [9,10]. However, the results of the model development process revealed distinct sets of features that led to the highest accuracies for the SVM, LR, and RF models. The most effective SVM model was a 3-feature model, including Fear, TUG_Cadence toward Chair, and 10MWT_std Swing Total, achieving accuracy, sensitivity, and specificity values of 0.86, 0.9, and 0.8, respectively. The top LR model also consisted of 3 features: Worried when Walking, Age, and CE_Balance_Thorax Linear Acc 2, with accuracy, sensitivity, and specificity of 0.71, 0.73, and 0.7, respectively. Lastly, the RF model, which achieved the highest accuracy among all three models, identified a top model with 2 features: Dual_Balance_Thorax Linear Acc 2 and TUG_Walk toward Cone, resulting in accuracy, sensitivity, and specificity values of 0.91, 0.82, and 1, respectively.

Table 4 presents the top fall risk assessment models using three sensor configuration approaches: single-sensor, double-sensor, and triple-sensor. For single-sensor models, the thorax and pelvic regions provided effective sensor locations across various combinations of 1–3 tasks. The triple-sensor models with sensors on the upper legs, lower legs/feet, and thorax achieved the highest accuracy of 0.91 using just the balance and TUG tests. Overall, balance tests and TUG, with or without cognitive dual-tasking, were most frequently included in the top models. The single thorax sensor model with balance, TUG, and STS tests produced a good accuracy of 0.91. This demonstrates the capability of a simple, single thorax-worn sensor setup to assess fall risk through a short battery of clinical tests. The triple-sensor models did not considerably improve accuracy over the best single-sensor models.

## 4. Discussion

Stroke Survivors (SS) often suffer from affected neuromusculoskeletal functions and reduced motor capabilities, increasing the risk of falling during routine tasks. Falls can lead to fatal injuries and serious health implications for such patients. Detection of fall-risk is crucial to ensure that necessary interventions are employed for those more prone to falling [4,7,21]. As current clinical fall risk evaluations rely on subjective scales and questionnaires, their outcomes may be biased, leading to errors in fall risk estimation. Our earlier studies have shown the benefits of using detailed motion analysis to identify lost functions in SS [22,23]. The use of motion data can lead to higher accuracy in predicting fall risk as compared to test scores from clinical tests [24]. Building upon our prior work, in this study, we provide a quantitative approach to predicting fall risk using machine learning approaches. We assessed the movement of 21 SS using 8 wearable motion sensors placed on their lower limbs, pelvis, and thorax as they performed balance tests, TUG, 10MWT, and STS, with/without motor-cognitive dual-task conditions. Using both motion data and clinical information, we extracted a robust set of 92 clinical and time domain kinematic features including accelerations, segment angles, temporal characteristics, and dual task costs. Supervised classification algorithms, namely SVM [25], LR [26], and RF [27,28] can be beneficial in predicting fall risk using motion data from instrumented clinical tests. In this study, we first distinguished between high and low fall-risk individuals and then through heuristic feature selection, identified optimal predictive subsets for each model. This comprehensive study demonstrates the feasibility of an objective, wearable sensor, and machine-learning-based approach to evaluate fall risk in stroke survivors, which in turn can assist clinicians in prescribing preventive interventions.

Among the three machine learning models, the RF classifier achieved the highest accuracy of 91% using just two predictive features (medio-lateral balance, and gait speed in TUG) and was derived from the motor-cognitive dual-task infused balance and TUG tests. The models presented in this study achieved high accuracies comparable to previous efforts that aimed to develop fall-risk prediction models for patients with a range of conditions (60–87%) that included older adults, SS, and multiple sclerosis, and total hip arthroplasty patients [27,28,29,30,31]. Furthermore, we ran two more models to identify the effect of having motion-related features and dual-task features in the process of fall risk assessment for SS. The model without motion-related features yielded accuracy, sensitivity, and specificity of 67%, 64%, and 70%, respectively. Also, the model without the dual-task features (only using the single-task features and clinical/demographic features) achieved accuracy, sensitivity, and specificity of 81%, 82%, and 80%, respectively. These results showed that the implementation of the motion-related features and dual-task paradigm improves the accuracy of the fall risk assessment model’s performance.

For the top RF model, the first feature was the dual-task balance thorax acceleration, which captured the medio-lateral trunk sway during standing with eyes open and simultaneous counting backwards. On the other hand, the TUG walk time consisted of measuring gait speed during the walk phase from the chair toward the cone. One of the reasons for high accuracies could be the infusion of dual tasking. Prior assessments of fall risk under divided attention conditions in the form of motor-cognitive dual-tasks have led to an increased fall-risk in patients of diverse neurological disorders, including stroke [8,17,18,32,33,34]. The dual-task challenges mobility and balance, unmasking deficits not apparent in single tasks, agreeing with findings from previous studies that evaluated the impact of dual tasks on movement [32,33]. The other two models used in this study included SVM and LR, which achieved moderately high accuracy between 71–86%. Overall, the machine learning approach proved highly capable of distinguishing between high and low fall-risk stroke survivors based on the defined battery of clinical assessment tasks.

Prior studies have shown different approaches for assessing fall risk using wearable sensors placed at different locations on the body [35], but less emphasis has been observed on determining the most efficient (least number of sensors for achieving high accuracy, as well as determining optimum placement locations) method for assessing fall risk. We investigated the prediction performance of the three machine learning models for features extracted from different configuration settings of wearable sensors, single, double, and triple sensors. An analysis of optimal sensor configurations and test batteries for fall risk assessment has been listed in Table 4. Three setups were compared: single-sensor models, double-sensor models, and triple-sensor models. For single sensor models, the thorax and pelvis regions provided effective sensor locations across various combinations of 1–3 clinical tests. Features from thorax and pelvic sensors during balance, TUG, and STS tests were the most predictive. This demonstrates that a simple, minimal setup with one inertial sensor on the thorax or pelvis can extract kinematic information to quantify fall risk. The triple-sensor models with additional sensors on the lower limbs did not lead to considerable improvements in accuracy over the best single-sensor models. The maximum accuracy achieved was 0.91 for both the single- and triple-sensor models. Thus, augmented sensor configurations appear to have diminishing returns for fall risk evaluation in stroke survivors. Between the balance test, TUG, 10MWT, and STS, the TUG and balance tests were most frequently included in the highest performing models. This affirms the specific utility of these standard clinical tests for assessing fall risk. TUG evaluates timed mobility skills while balance tests assess static steadiness. Taken together, the results indicate that a single thorax-worn sensor capturing dual-task TUG, dual-task balance, and dual-task STS metrics can provide robust fall risk assessment comparable to more complex multi-sensor systems. This result highlights the high significance of implementing a motor-cognitive dual-task paradigm for fall risk assessment in stroke survivors. Finally, the single-sensor configuration could be replaced with a smartphone that has IMU technology embedded to make the risk assessment procedure much cheaper, easier, and accessible [19,36].

Recent efforts in the research community aim toward the exploration of data-centric methods for objectively evaluating the fall risk of patients using accessible and portable wearable technologies [35]. In our study, the single thorax sensor model with balance, TUG, and Sit-to-Stand tests produced an accuracy of 0.91. This configuration offers an accessible and low-cost approach to screen for fall risk using three standard clinical tests using a single IMU-based wearable sensor to capture mobility performance and postural sway without ceiling effects. Moreover, the entire protocol can be completed in under 10 min using the TUG task, which is familiar, safe, and requires minimal space. The high accuracy also demonstrates potential utility for clinics, as well as for at-home assessments with limited resources [37]. Specifically, this can allow for conducting periodic home-based screening to identify changes in fall risk over time, as well as to assess the progress over the course of rehabilitation programs [21,38]. The simple low-cost system could also extend fall risk screening to areas with limited healthcare access. Ultimately, the obtained quantitative fall risk scores could assist therapists in prescribing appropriate interventions. Overall, the single sensor configuration with TUG, balance and sit-stand offers an objective and practical approach to evaluate and monitor fall risk in stroke survivors.

Although our approach shows promise in efficiently predicting fall risk in SS using wearable sensors and machine learning models, it is important to acknowledge the limitations of this research. The relatively small sample size of 21 participants may restrict the generalizability of the findings, emphasizing the need for larger and more diverse cohorts to validate and refine the models. Once validated using a much larger sample size, this model could enable regular fall risk monitoring and prevention in community settings. Further research should evaluate real-world implementation across diverse mobility levels, living environments, and clinical workflows. Patient perspectives on the usability of the proposed model should also be gathered. Our study primarily included high-functioning SS who could walk unassisted for 10 m, potentially limiting the applicability of the results to those with more severe mobility impairments. Further investigations should encompass a broader range of stroke disability levels. Additionally, the study focused on specific sensor configurations and feature extraction methods, leaving room for exploring alternative setups and extraction techniques to improve model accuracy and reliability. Particularly, frequency domain features could provide complementary information beyond the time-based characteristics assessed here as demonstrated in recent spectral biomarker research [9,10]. Despite these constraints, this research underscores the potential of wearable sensors and the use of machine-learning-based approaches in conducting fall risk assessment for SS and provides a foundation for future studies to develop efficient tools for conducting fall risk assessment.

## 5. Conclusions

This study presents a promising approach to fall risk assessment in stroke survivors using wearable sensors and machine learning models. The results demonstrate the feasibility of using inertial sensors during clinical assessment tasks along with machine learning algorithms to objectively quantify and classify fall risk. The Random Forest model achieved 91% accuracy in distinguishing high versus low fall risk individuals using only two motion features related to balance and walking. Analysis of optimal sensor configurations revealed that a single thorax-worn sensor can effectively assess fall risk through a short battery of balance, TUG, and sit-stand tests. These findings underscore the potential of a quantitative, wearable sensor-based method to evaluate fall risk in stroke survivors compared to current subjective scales. With further validation on larger cohorts, the developed approach could assist clinicians in prescribing preventive interventions through objective screening. This could help avoid injurious falls and enhance mobility in stroke survivors. The single thorax-worn sensor model provides a pragmatic configuration for clinical and in-home assessment. However, some limitations should be noted. The modest sample size may restrict generalizability and model optimization. Testing across a wider range of stroke disability levels is also needed. There are opportunities to explore different sensing modalities, alternate sensor placements, and more sophisticated analytics. Overall, this research provides an important step toward developing portable, accurate tools for fall risk evaluation in stroke survivors. Quantitative screening can support patient-specific rehabilitation to improve balance and mobility. Wearable sensor systems with machine learning have a strong potential to make fall risk assessment in neurological disorders more objective, enhancing clinical decision-making and patient outcomes.

## Figures and Tables

**Table 1:** Lists of tasks within the experimental test battery along with their descriptions.

Test Battery Tasks (with & without Cognitive Load)	Description
Balance Test	Stand as still as possible with open/closed eyes on a firm surface for 30 s
Timed Up and Go (TUG)	Stand up from a normal chair, walk for 3 m, return, and sit on the chair
10 Meter Walk Test (10 MWT)	10 m straight walk without a turn
Sit to Stand (STS)	Standing up and sitting down on a chair 5 times
Motor-cognitive Dual-tasks	Repetition of the five above tasks while counting backward from 200 by the step of 10

**Table 2:** The extracted features from each test implemented as the initial pool of features in the development of a machine learning model to predict the risk of fall. Note that Acc 1: acceleration toward the front side of the participant, Acc 2: acceleration toward the right side of the participant, Acc 3: acceleration toward down, Acc 4: resultant acceleration, std: standard deviation, Ang Vel: Angular Velocity, CE: closed eyes.

	Test Battery	Features
**Functional Tests**	Balance Test (open eyes)	Balance_Thorax Linear Acc 1, Balance_Thorax Linear Acc 2, Balance_Thorax Linear Acc 3, Balance_Thorax Linear Acc 4, Balance_Pelvic Linear Acc 4, Balance_Right Thigh Linear Acc 4, Balance_Left Thigh Linear Acc 4
	Balance Test (closed eyes)	CE_Balance_Thorax Linear Acc 1, CE_Balance_Thorax Linear Acc 2, CE_Balance_Thorax Linear Acc 3, CE_Balance_Thorax Linear Acc 4, CE_Balance_Pelvic Linear Acc 4, CE_Balance_Right Thigh Linear Acc 4, CE_Balance_Left Thigh Linear Acc 4
	Timed Up and Go (TUG)	TUG_Time, TUG_Sit to Stand, TUG_Walk toward Cone, TUG_Turn around the Cone, TUG_Walk toward Chair, TUG_Turn and Sit, TUG_Steps toward Cone, TUG_Steps toward Chair, TUG_Cadence toward Cone, TUG_Cadence toward Chair
	10 Meter Walk Test (10MWT)	10MWT_Walk Time, 10MWT_Step, 10MWT_Cadence, 10MWT_mean Swing Total, 10MWT_std Swing Total, 10MWT_Single Support, 10MWT_Stride Duration, 10MWT_std Stride Duration
	Sit to Stand (STS)	STS_Time, STS_Mean Sit to Stand, STS_Mean Stand to Sit, STS_Thorax Ang Vel, STS_Pelvic Ang Vel, STS_std STS, STS_std Sit to Stand, STS_std Stand to Sit, STS_std Thorax Ang Vel, STS_std Pelvic Ang Vel
	Dual-Task	All the features for the 5 above tests calculated for dual-task tests.
**Clinical Information**		BMI, Gender, Age, Feel Unsteady, Worried when Walking, Joint Pain, Number Neurological Disease beside Stroke, Fear of Fall (FES Questionnaire)

**Table 3:** Descriptive statistics for measures across the different levels of fall risk for stroke survivors. (Note: M and F denote male and female participants).

Parameter	Faller (N = 11)	Non-Faller (N = 10)	Total (N = 21)
Gender	4 M, 7 F	7 M, 3 F	11 M, 10 F
Age (year)	64.1 (12.3)	67.6 (7.1)	66 (10)
Height (cm)	172.7 (8)	175 (9.1)	173.8 (8.4)
Weight (kg)	84 (13.3)	90.8 (16.2)	86.3 (14.7)
BMI (kg/m^2^)	28.1 (3.5)	29 (4.7)	28.5 (4)
Poor Vision	1	1	2
Joint Pain	8	6	14
Feel Unsteady while Walking	9	6	15
Worried of Falling when Walking	7	3	10
Lack of Normal Cognitive Function to be Independent with ADLs	0	0	0
Joint Replacement	3	3	6
Short FES-I (out of 28)	13.1 (5.1)	9.1 (3)	11.2 (4.6)

**Table 4:** The top predictive models for the three simplest configurations and their relevant characteristics and performance.

Sensor Configuration	Sensor Location	Test Battery	Features	Accuracy	Sensitivity	Specificity
Single-sensor	Thorax	Balance with Dual-task	Dual_Balance_Thorax Linear Acc 2	0.86	0.91	0.8
	Thorax	TUG with Dual-task	Dual_TUG_Turn and Sit	0.76	0.73	0.8
	Thorax	Balance with Dual-task, TUG with Dual-task, STS	Dual_ Balance_Thorax Linear Acc 2, Dual_TUG_Turn and Sit, STS_Time, Age, Dual_STS_Thorax Ang Vel	0.91	0.91	0.9
	Pelvic	STS	Dual_STS_std Pelvic Ang Vel	0.76	0.73	0.8
	Pelvic	TUG with Dual-task, Balance, STS with Dual-task	Dual_TUG_Turn and Sit, Dual_STS_std Pelvic Ang Vel, Fear (FES), Balance_Pelvic Linear Acc 4	0.81	0.82	0.8
Double-sensor	Thorax and Pelvic	Balance w/o Dual-task	Dual_Balance_Thorax Linear Acc 2, Balance_Pelvic Linear Acc 4, Balance_Thorax Linear Acc 4	0.91	0.82	1
	Upper Legs	TUG with Dual-task	Dual_TUG_Turn and Sit	0.76	0.73	0.8
	Lower Legs or Feet	TUG and 10MWT	10MWT_mean Swing Total, TUG_Cadence toward Chair	0.81	0.82	0.8
Triple-sensor	Upper Legs and Thorax	Balance with Dual-task, TUG	Dual_Balance_Thorax Linear Acc 2, TUG_Walk toward Cone	0.91	0.82	1
	Lower Legs or Feet and Thorax	Balance and STS with Dual-task, TUG	Dual_Balance_Thorax Linear Acc 2, TUG_Walk toward Cone, Dual_STS_Thorax Ang Vel	0.91	0.91	0.9

## Data Availability

The raw data supporting the conclusion of this article will be made available by the corresponding author on reasonable request.
